# VOICES OF OUR ELDERS: ATTITUDES, BELIEFS, AND PERSPECTIVES ABOUT RESEARCH IN MINORITY OLDER ADULTS

**DOI:** 10.1093/geroni/igac059.1863

**Published:** 2022-12-20

**Authors:** Trudy Gaillard, Donna Shambley-Ebron, Giovanni Garcia, Ryan Romero, Donna Neff, Phildra Swagger, Dawn Gardier, Fern Webb

**Affiliations:** Florida International University, Miami, Florida, United States; University of Cincinnati, Cincinnati, Ohio, United States; Florida International University, Miami, Florida, United States; Florida International University, Miami, Florida, United States; University of Central Florida, Orlando, Florida, United States; University of Central Florida, Orlando, Florida, United States; Florida International University, Miami, Florida, United States; University of Florida (UF) College of Medicine, Jacksonville, Jacksonville,, Florida, United States

## Abstract

The U.S. Census Bureau projects that the numbers of adults 65 and older will double from 46 million in 2020 to 90 million by 2050, thus representing the fastest growing segment of the population. However, older adults, especially those from minority groups, remain underrepresented in clinical research. It is imperative to understand what older adults believe about research and research participation to enhance recruitment efforts. The aim of this presentation is to present preliminary findings from our qualitative study which explored the attitudes, beliefs, and perspectives of older minority adults regarding research and research participation. We conducted 12 focus groups via Zoom, in South Florida with minority adults (African American, Caribbean, Hispanic) over the age of 65 (N=49). An interview guide was used to query the participants about their attitudes, beliefs, and perspectives of research and research participation. Focus groups were video-recorded and transcribed. NVivo software was used for data management and analysis. We found that participants: 1) thought research was necessary to expand understanding and knowledge of health conditions; 2) stated research should be conducted by trusted scientific institutions; 3) relied heavily on their adult children for advice regarding research participation; 4) expressed reluctancy regarding invasive procedures; and 5) were influenced by personal experiences when considering research participation. Our preliminary findings suggest that older minority adults believe in the value of research, however, may be hesitant about participating. We propose continued strategies aimed at increasing engagement of minority older adults into health research.

